# Lipid overload during gestation and lactation can independently alter lipid homeostasis in offspring and promote metabolic impairment after new challenge to high-fat diet

**DOI:** 10.1186/s12986-017-0168-4

**Published:** 2017-02-20

**Authors:** Laís Angélica de Paula Simino, Thaís de Fante, Marina Figueiredo Fontana, Fernanda Oliveira Borges, Márcio Alberto Torsoni, Marciane Milanski, Lício Augusto Velloso, Adriana Souza Torsoni

**Affiliations:** 1 Laboratory of Metabolic Disorders, Faculty of Applied Sciences, University of Campinas -UNICAMP, Limeira, São Paulo Brazil; 2 Laboratory of Cell Signaling, Faculty of Medical Sciences, University Of Campinas - UNICAMP, Campinas, São Paulo Brazil

**Keywords:** microRNAs, Maternal obesity, Metabolic programming, NAFLD

## Abstract

**Background:**

Nutritional status in early life is critically involved in the metabolic phenotype of offspring. However the changes triggered by maternal consumption of high-fat diet (HFD) in pre- or postnatal period should be better understood. Here we evaluated whether maternal HFD consumption during gestation and lactation could differently affect liver miR-122 and miR-370 expression leading to metabolic damages observed in offspring. Moreover, we investigate whether early overnutrition program offspring to more harmful response to HFD in later life.

**Methods:**

Female mice were fed either a standard chow (SC) diet or a HFD three weeks before and during mating, gestation and/or lactation. Offspring were evaluated on the delivery day (d0), in a cross-fostering model at day 28 (d28) and in adult life, after a re-challenge with a HFD (d82).

**Results:**

In vitro analysis using liver cell line showed that palmitate could induced decrease in miR-122 and increase in miR-370 expression. Newborn pups (d0) from obese dams showed a decrease in lipid oxidation markers (*Cpt1a* and *Acadvl*), an increase in triacylglycerol synthesis markers (*Agpat* and *Gpam*), as well as lower miR-122 and higher miR-370 hepatic content that was inversely correlated to maternal serum NEFA and TAG. Pups fostered to SC dams presented an increase in body weight and *Agpat*/*Gpam* expression at d28 compared to pups fostered to HFD dams and an inverse correlation was observed between miR-122 hepatic expression and offspring serum TAG. In adult life (d82), the reintroduction of HFD resulted in higher body weight gain and hepatic lipid content. These effects were accompanied by impairment in lipid and glucose metabolism, demonstrated by reduced *Cpt1a*/*Acadvl* and increased *Agpat*/*Gpam* expression, lower glucose tolerance and insulin sensitivity.

**Conclusion:**

Our data suggest that both gestational and lactation overnutrition results in metabolic changes that can permanently alter lipid homeostasis in offspring. The presence of fatty acids in maternal blood and milk seem to be responsible for modulating the expression of *miR-122* and *miR-370*, which are involved in liver metabolism. These alterations significantly increase susceptibility to obesity and ectopic lipid accumulation and lead to a more harmful response to HFD in offspring.

**Electronic supplementary material:**

The online version of this article (doi:10.1186/s12986-017-0168-4) contains supplementary material, which is available to authorized users.

## Background

Nutritional status in early or pre-natal life is critically involved in susceptibility to cardiovascular and metabolic diseases development, such as hypertension, dyslipidemia, hyperglycemia and obesity [1, 2], events which characterize the metabolic syndrome. Non-alcoholic fatty liver disease (NAFLD) has been considered the hepatic manifestation of this condition and, additionally, is one of the most common causes of liver diseases worldwide [3–5]. The liver plays a central role in modulating glucose homeostasis and lipid metabolism, and NAFLD is caused by an imbalance in lipid metabolism pathways involved in triacylglycerol synthesis, export, delivery, and oxidation [6, 7].

Recent studies demonstrated that increased adiposity, hepatic insulin resistance and ectopic fat accumulation have been associated with maternal body weight gain and exposure to a high-fat diet (HFD) along critical phases of development, such as gestation and lactation [8–11]. Moreover, offspring exposed to a HFD *in utero* and through lactation presents impaired hepatic mitochondrial function and up-regulation of lipogenesis, factors that may contribute to the development of NAFLD and to the progression to a more aggressive liver disease, the non-alcoholic steatohepatitis (NASH) [12].

It is known that lipids can act as signaling molecules and transcriptional activators, and hepatic gene transcription regulation by fatty acids was first reported in 1990s [11, 13, 14]. Saturated fatty acids (SFA), particularly, induce hypothalamic inflammation, endoplasmatic reticulum stress, deleterious effects on blood lipid and lipoprotein profile and, in the liver, can bind to nuclear receptors of transcriptional factors involved in lipid homeostasis and induce lipid droplet accumulation [15, 16].

Perinatal exposure to fatty acids overload, specially SFA, may trigger epigenetic mechanisms that regulate genes involved in lipid sensing and metabolism [11]. MicroRNAs (miRNAs) are epigenetic modulators of gene expression that acts as mRNA silencers, and their regulation are reported to be involved in almost all biological processes in animals [17, 18]. In contrast, studies have shown that multiple factors can interfere in miRNA expression, such as toxic, chemical and environmental agents and also dietary components [19]. *miR-122* and *miR-370* participate in the regulation of hepatic lipid metabolism [20–26]. *miR-122* is predicted to modulate lipogenic genes and to be potentially targeted by *miR-370* which, in turn, can directly bind to carnitine palmitoyltransferase 1α (*Cpt1a*) gene [20, 21].

In a recent study, we showed that maternal HFD consumption during pregnancy and lactation leads to a decreased *miR-122* and increased *miR-370* expression in the liver of recently weaned mice [25]. These miRNAs alterations occurred concurrently with higher expression of lipogenic genes (*Gpam*, *Agpat* and *Scd1*) and lower expression of genes related to fatty acid oxidation (*Cpt1a* and *Acadvl*) [25]. However, although our previous results reports that *miR-122* and *miR-370* may participate in the genesis of metabolic damage associated to fatty liver [25], it is not possible to assign the role of gestational or lactational periods to the effects observed in offspring from obese dams and literature data concerning these phenomena are very controversial.

Using a cross-fostering model, Oben and co-workers (2010) showed that lean offspring suckled by obese dams presents increased body weight and food consumption, along with metabolic complications evidenced by increased insulin and leptin levels in plasma and development of NAFLD in adulthood [27]. In contrast, other studies suggest that health status in adulthood is primarily determined by the conditions under which an organism develops in the womb. Gniuli and colleagues [28] showed that exposure to a HFD *in utero* may lead offspring to a type 2 diabetes phenotype, which could even be transmitted to the progeny. Moreover, maternal consumption of HFD during pregnancy was reported to cause a dysregulation in triglyceride metabolism and, in adipose tissue, to lead to increasing in leptin and suppression of adiponectin levels through epigenetic modifications, leading offspring to a metabolic syndrome-like phenomenon [29].

Importantly, it was previously shown that the metabolic alterations in offspring from HFD fed dams during gestation and suckling period, such as leptin and insulin resistance and ectopic fat accumulation in the liver, persists into adult life, even when they are maintained on a healthy standard chow diet after weaning [10]. However, the molecular mechanism associated to hepatic lipid metabolism modification and the development of fatty liver in adult offspring from HFD fed dams still need to be clarified.

Additionally, another question is still to be answered: which stimulus present at gestational and/or lactational period would be responsible for triggering metabolic programming of offspring from obese dams? The majority of experimental protocols used to study the foetal programming by maternal obesity consist of inducing weight gain through a HFD consumption. During pregnancy, nutrients are transported from maternal circulation to the fetuses via the placenta and, at suckling periods, maternal diet directly affects milk composition [30–34]. Therefore, we hypothesized that the excessive maternal lipids, particularly SFA, could be the trigger for the miRNAs modulation that we earlier reported in the liver of offspring [25].

Thus, the aim of the present study was to test the hypothesis that maternal high fat diet could directly modulate *miR-122* and *miR-370* expression and to investigate the independent contribution of pregnancy and lactation to the altered hepatic lipid metabolism observed in young offspring from obese dams. Additionally, we also evaluated whether this metabolic programming would persist to adulthood and, moreover, whether offspring from HFD-fed dams would present increased metabolic complications after a re-challenge to nutritional overload in adult life.

## Methods

### In vitro analysis

HepG2 human hepatocyte cell line was kindly provided by Dr. Gabriel Forato Anhe, from Farmacology Department of Medical Sciences Faculty of State University of Campinas. Hepa1c1c7 mouse hepatocyte cell line was purchased from BCRJ (Rio de Janeiro Cell Bank, Brazil). HepG2 was maintained in DMEM (Dulbecco’s modified Eagle’s Medium) and Hepa1c1c7 in α-MEM (Minimum Essential Medium Eagle without nucleosides), both supplemented with 10% FBS, 100U/mL penicillin and 0,1 mg/mL streptomycin, incubated at 37 °C in 5% CO2. Experiments were performed between passages 5 and 40.

Cells were grown as monolayers at a density of 4 × 10^5^ cells/mL and two days after seeding, the cultured cells were treated for 6 h with Palmitate (Sigma Aldrich) 500 μM or Palmitate vehicle (NaOH + BSA, 3:1) as a control.

### Animals

**Fig. 1:**
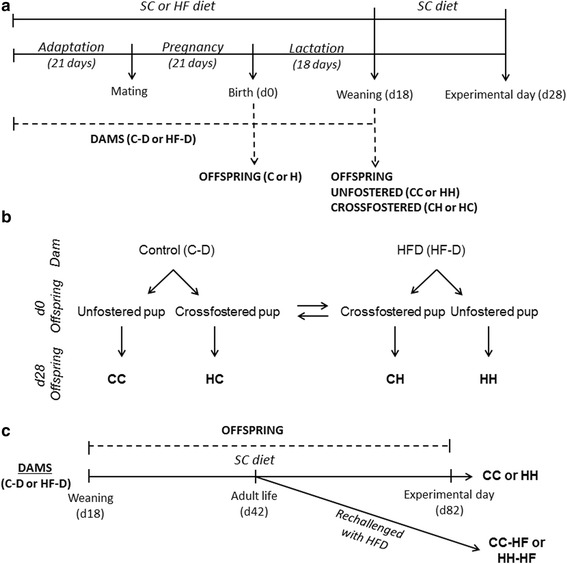
Experimental Protocol. Experimental protocol followed to investigate the effect of maternal overnutrition on offspring metabolism, during gestation or lactation. Offspring of control (C-D) and HFD (HF-D) dams were evaluated at d0 (C and H) and d28 (CC, CH, HH and HC) (**a**). Details of the protocol followed to obtain cross-fostered groups (**b**). Experimental protocol followed to evaluate the metabolic complication in offspring after re-challenge to adult life in nutritional overload at d82 (CC, CC-HF, HH, and HH-HF) (**c**)

Five weeks old virgin female and male Swiss mice (*Mus musculus*) were obtained from the Animal Breeding Center at the University of Campinas (Campinas, São Paulo, Brazil) for mating. The general experimental design is illustrated in Fig. 1.

Female mice were randomly fed either HFD or standard chow diet (SC) *ad libitum* for three weeks before mating (adaptation period). HFD was prepared according to Benatti et al. [25] (Table 1).

**Table 1:** Nutritional composition of the standard chow and experimental diets offered to mice during adaptation, gestation, lactation and post-natal life

	SC Diet^a^	Growth HFD	Maintenance HFD
Net Protein (g %)	20^b^	23,3^c^	16,7^c^
Fat Content (g %)	4^d^	24^e^	24^e^
Carbohydrates (g %)	65,95^f^	42,1^f^	49,3^f^
Fiber (g %)	5	5,55	5
Mineral Mix (g %)	3,5	3,5	3,5
Vitamin Mix (g %)	1	1	1
Choline (g %)	0,25	0,25	0,25
Cystine (g %)	0,3	0,3	0,25
Total	100	100	100
Energy (KJ/g)	14,6	19,3	19,3

^a^NUVILAB® Cr-1; Nuvital

^b^vegetal protein - from wheat and corn (added lysine and methionine)

^c^animal protein - casein

^d^soy oil

^e^soy oil and lard

^f^starch and saccharose

Adult males were fed only SC during the adaptation period. Mating was performed by housing females for three days with males. Pregnancy was confirmed by examination of vaginal smears for the presence of sperm.

Pregnant females were maintained in individual polypropylene micro-isolators, at 22 ± 1 °C and lights on from 06.00 to 18.00 h. The same diet offered before mating was maintained during gestation period. The study was performed following two protocols: one of them was used to evaluate the effect of maternal overnutrition on offspring metabolism, during gestation or lactation; the other protocol was used to evaluate the metabolic complication in offspring from high fat dams after re-challenge to adult life in nutritional overload (Fig. 1).

To the first protocol on day 0 (d0, first post-partum day), litters were divided in two groups according to maternal diet: offspring from female mice fed SC were called C and those from female mice fed HFD were called H. Some litters were euthanized and used for d0 analysis. The remaining litters were adjusted to eight pups each to ensure a standard litter size per mother and were followed until d28 (Fig. 1a).

To study the effect of maternal exposure to HFD on offspring metabolism, during gestation or lactation separately, we carried out the cross-fostering protocol. At d0, individual pups from the litters were randomly assigned to two different foster conditions: (a) un-fostered (i.e. the pups were fed by their own mother) and (b) cross-fostered (i.e. the pups were fostered by a mother from the other group). Four groups were obtained: CC and HH, in which pups were exposed to SC or HFD during gestation and lactation, respectively, and HC and CH, in which pups were exposed to HFD only during gestation or only during lactation, respectively (Fig. 1b). All pups were weaned on day 18 and separated according to sex. Only males were used to perform the analysis. The cross-fostered (CH and HC) and un-fostered (CC and HH) groups were fed exclusively an SC diet after weaning until experimental day 28 (d28).

To the second protocol the un-fostered pups from CC and HH groups were maintained on SC diet after weaning until day 42 (d42). Part of the litters remained on standard chow diet until day 82 (CC and HH) and the other part were re-challenged with a HFD until day 82 (CC-HF and HH-HF) (Fig. 1c).

Females in adaptation, gestation or lactational periods were fed growth HFD to ensure an adequate protein supply, while offspring exposed in adult life (CC-HF and HH-HF) were fed maintenance HFD (Table 1).

The total number of animals used in each experiment is mentioned in the figure legends.

### Body composition

Body weight was measured weakly for dams and pups. To estimate the body fat in d0 groups, we measured naso-anal length of pups, and Lee Index of Obesity (LIO) was calculated using the formula: body weight (g) (1/3) / naso-anal length (cm). To measure adiposity in dams, d28, d42, and d82 groups, white adipose tissue (epidydimal and retroperitoneal) was collected and weighted after sacrifice, and the percentage of adiposity normalized by total body weight was calculated.

### Food intake

Food intake was estimated for dams (at gestational day 12 and lactation day 15), d28, d42, and d82 groups during 24 h over a period of four days. The average was considered as food intake (Kcal/d).

### Biochemical analysis

To evaluate triglycerides (TAG) and cholesterol (CHOL) levels, blood samples were collected on the experimental days after overnight fasting. By enzymatic colorimetry, serum aliquots were used to measure the levels of TAG and cholesterol.

To evaluate fasting glucose, blood samples were collected after overnight fasting by decapitation or from the tail. Glycaemia was determined on an Accu-Chek Performa glucometer.

To determine serum insulin and leptin, blood samples were collected on the experimental days after overnight fasting. Serum insulin were determined using Rat/Mouse Insulin Kit from Millipore and serum leptin were determined using Mouse Leptin ELISA kit.

Hepatic total lipids content was evaluated as proposed by Folch and colleagues [35].

### Intraperitoneal Glucose, Insulin and Pyruvate Tolerance Test (GTTip, ITTip and PTTip)

Tolerance tests were performed in adult offspring exposed to a HFD and the same animals were used for all tests, with an interval of at least one week between each test. Glycaemia was determined in an Accu-Chek Performa Glucometer (Roche Diagnostics, Basel, Switzerland). Pyruvate tolerance test was performed to more directly assess the role of HFD in hepatic glucose homeostasis in adult offspring from obese dams.

For the glucose (GTT) and insulin (ITT) tests, mice were starved for 12 h, fed for h and starved for 4 additional hours before intraperitoneal (IP) injections. For GTT, a solution of 25% D-glucose was used and each animal was injected with 1 g/kg of glucose. Glycaemia was measured at 0, 15, 30, 60, 90 and 120 min after glucose administration. For ITT, recombinant regular insulin (1,5 UI/kg, Humulin®, Eli Lilly and Company, EUA) was administrated by IP injection and glycaemia was measured at 0, 3, 6, 9, 12 and 15 min after injection.

For the pyruvate (PTT) test, mice were starved for 12 h and fed for 2 h, and the test was performed in fed state. IP injection of a pyruvate solution (20%) was administered in a dose of 2 g/kg and glycaemia was measured at 0, 15, 30, 60, 90 and 120 min after pyruvate administration.

In both GTT and PTT, results are presented as area under curve (AUC) of glycaemia *vs*. time, above each individual baseline. In ITT, results are presented as kITT, the constant for the glucose clearance rate, calculated using the formula 0,693/t1/2.

### Quantitative Real-Time PCR (qRT-PCR)

Total hepatic RNA was extracted from liver (150 mg), using TRIzol reagent according to the manufacturer’s recommendations and quantified using NanoDrop ND-2000. Reverse transcription was performed with 3 μg of total RNA, using a high-capacity cDNA reverse transcription kit (Thermo Fisher Scientific). Relative expression was determined using a Taqman detection system and primers for the target genes: *Agpat1* (Mm 00479699_g1*)*; *Acadvl* (Mm 00444293_m1); *Cpt1a* (Mm 01231183_m1); *Gpam* (Mm 00833328_m1) for liver analysis. Actin beta (*β-Actin,* Mm 02619580_g1) was used as the endogenous control (Thermo Fisher Scientific).

Each PCR contained 20 ng of complementary DNA. Real-time PCR was performed on an ABI Prism 7500 Fast platform. Data were analyzed using the sequence detection system 2.0.5 and expressed as relative values determined by the comparative threshold cycle (Ct) method (2 − ΔΔCt) according to the manufacturer’s recommendation.

### Purification and quantification of miR

The miR content was extracted and purified from the Hepa1c1c7 and HepG2 cells and from the liver (150 mg) of the mice using the mirVana miRNA isolation kit (Thermo Fisher Scientific) according to the manufacturer’s instructions. The relative expression of *miR-122* and *miR-370* (ID 002245 and ID 002275, respectively, Thermo Fisher Scientific) was determined using primers with a TaqMan detection system and *U6 spliceosomal RNA* (ID 001973, Thermo Fisher Scientific) as endogenous controls. Gene expression was accessed by real-time PCR performed on the ABI Prism 7500 Fast platform and analyzed according to 2.5.

### Statistical analysis

Numeric results are expressed as means with their standard errors of the indicated number of experiments. Student’s *t*-test was used for unpaired samples and analysis of variance (ANOVA) for multiple comparisons. A post-hoc test (Bonferroni) was used to determine a significance level of *p* ≤ 0.05. Pearson’s correlations were used to determine the relation between miR-122 expression and serum TAG or NEFA and a linear regression ± 95% confidence interval analysis were performed. In all cases, the statistical significance was set at *p* ≤ 0.05. The statistical analyses used in each table and graph are specified in the respective legend.

## Results

### In vitro hepatocytes treatment with palmitate alters miR-122 and miR-370 levels

**Fig. 2:**
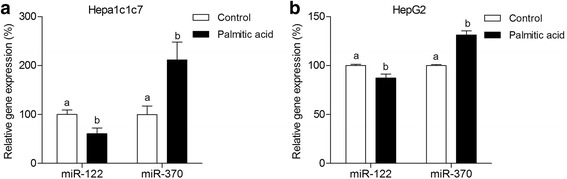
Fatty acid exposure induces miRNAs modulation in mouse and human liver cell lines. MicroRNA level (qRT-PCR) - *miR-122* and *miR-370* - from Hepa1c1c7 mouse hepatoma cell line (1x10^8^cells/mL) (**a**) and HepG2 human hepatoma cell line (1x10^8^cells/mL) (**b**) 6hs after exposure to palmitic acid (500uM). For relative gene expression analysis, U6snRNA was used as endogenous control. Values are means (*n* = 3–6) + - SEM. Student's *t*-test was used to compare Control and Palmitic Acid groups. Different letters indicate statistical significance between groups (*p* ≤ 0.05)

To test the hypothesis that fatty acids could modulate the expression of hepatic *miR-122* and *miR-370*, we performed in vitro analysis in mouse (Hepa1c1c7) and human (HepG2) hepatoma cell lines treated with palmitate. The treatment lead to a decreasing in *miR-122* (among 13 to 39%) and an increasing in *miR-370* levels (among 31 to 114%; Fig. 2a and b, respectively), indicating that excessive fat could alter the expression of these miRNAs in liver.

### Maternal HFD consumption in gestational period leads newborn offspring to altered miRNAs and lipid-related gene expression in the liver

Next, our goal was to evaluate in which moment - pregnancy or lactation - maternal excessive lipids would be responsible for the modulation of the miRNAs in offspring. For this purpose, we evaluated pups from HF-D and C-D in the day of delivery (d0).

**Fig. 3:**
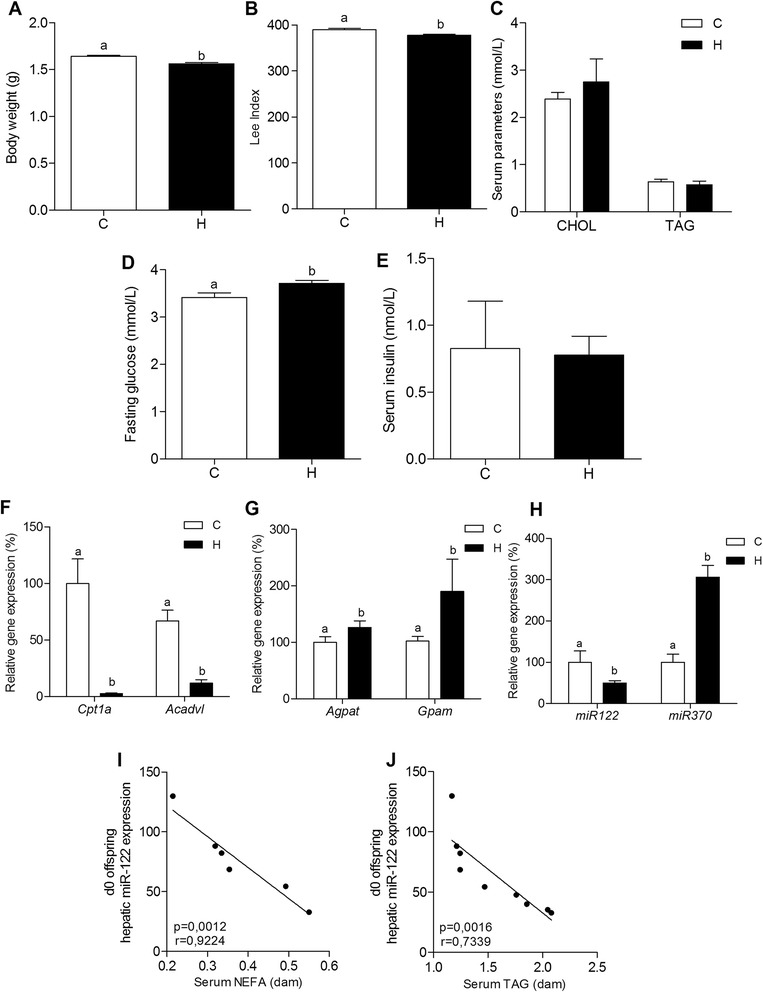
Maternal HFD consumption at gestation period leads newborn offspring to altered miRNAs and lipid metabolism-related gene expression. Body weight (**a**), lee index of obesity (LIO) (**b**), serum lipids (CHOL and TAG - **c**), fasting glucose (**d**) and serum insulin (**e**), mRNA levels (qRT-PCR) of hepatic *Cpt1a* and *Acadvl* (**f**), and *Agpat* and *Gpam* (**g**), microRNA level (qRT-PCR) of hepatic *miR-122* and *miR-370* (**h**) from newborn offspring from C and H groups. Correlation analysis between hepatic *miR-122* from d0 offspring vs. serum NEFA (**i**) and TAG (**j**) from dams. For relative gene expression analysis, β-Actin and U6snRNA were used as endogenous controls. Values are means (*n* = 5–12) + - SEM. Student's *t*-test was used in all analyses to compare C and H groups. Different letters indicate statistical significance between groups (*p* ≤ 0.05)

HFD consumption lead dams to alterations in body composition, impaired serum parameters and hepatic total lipids accumulation at gestational period (Additional file 1: Figure S1). Mice from dams fed HFD during gestation (H) showed lower body weight and LIO (Fig. 3a and b, respectively) in comparison to offspring from control dams (C). The serum levels of CHOL and TAG in C and H groups were very similar (Fig. 3c). However, we observed that H presented higher fasting glucose levels (9% more than C) immediately after birth, but the same insulin levels compared to C (Fig. 3d and e, respectively).

Mice from H group had reduced *Cpt1a* (38.5-fold) and *Acadvl* (5.6-fold; Fig. 3f) gene expression. An increase in *Agpat* (1.3-fold) and *Gpam* (1.9-fold; Fig. 3g) gene expression was also observed in offspring of obese dams.

Moreover, maternal consumption of a HFD during gestation provoked a decrease in *miR-122* (50%) and an increase in *miR-370* (206%) expression (Fig. 3h). Interestingly, liver *miR-122* expression in newborn offspring was inversely correlated with maternal serum TAG levels (*p* = 0.0016 - Fig. 3i).

### Maternal HFD consumption in gestational or suckling periods independently alters miR-122 and miR-370 and lipid-related gene expression in the liver of recently weaned offspring

To evaluate whether hepatic modulation of *miR-122* and *miR-370* by maternal HFD at gestational period would persist after weaning and to investigate if maternal milk would exert a similar effect, we fostered pups after birth.

**Fig. 4:**
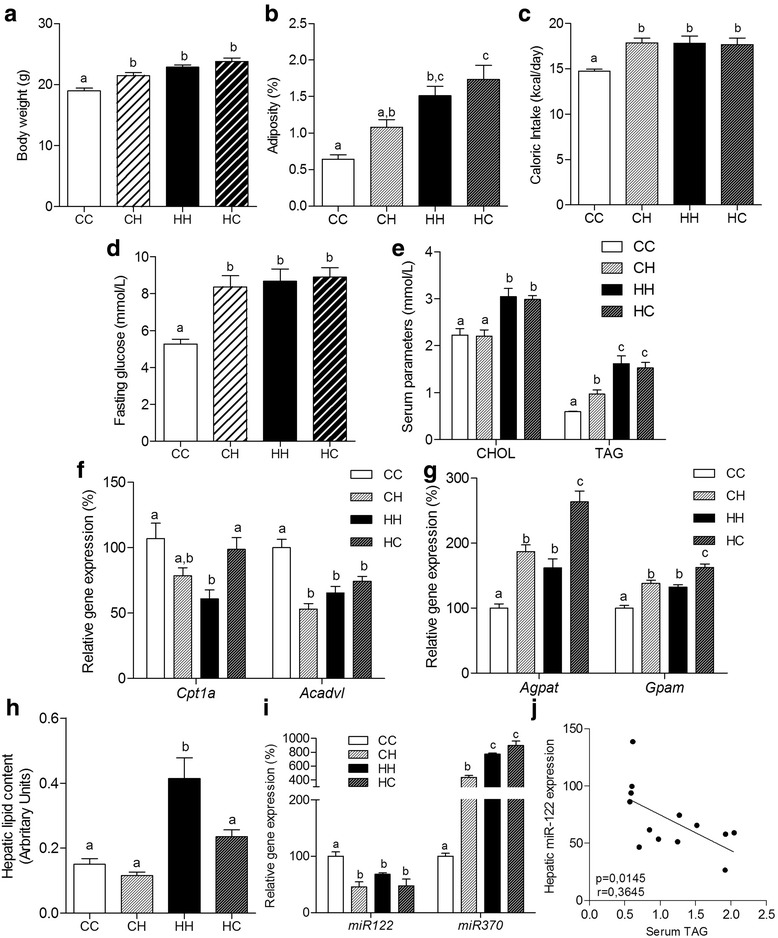
Maternal HFD consumption at gestation or lactation independently alters miRNAs and lipid-related gene expression. Body weight (**a**), adiposity (**b**), caloric intake (**c**), fasting glucose (**d**) and serum lipids (CHOL and TAG - **e**), mRNA levels (qRT-PCR) of hepatic *Cpt1a* and *Acadvl* (**f**), and *Agpat* and *Gpam* (**g**), hepatic total lipid content (**h**), microRNA level (qRT-PCR) of hepatic *miR-122* and *miR-370* (**i**), correlation analysis between hepatic *miR-122* and serum TAG (**j**) from recently weaned unfostered offspring (CC and HH) and crossfostered offspring (CH and HC) at d28. For relative gene expression analysis, β-Actin and U6snRNA were used as endogenous controls. One-way ANOVA was used in all analyses to compare CC, CH, HH and HC groups. Values are means (*n* = 5–8) + - SEM. Different letters indicate statistical significance between groups (*p* ≤ 0.05)

The alterations observed in body composition and serum biomarkers of HFD-fed dams at gestation persisted to lactation period (Additional file 1: Figure S1). At d28, we observed an increase in body mass in pups gestated, suckled or both by dams fed HFD compared to those gestated and suckled by control dams, since CH, HH, and HC showed a higher body weight than CC (Fig. 4a). However, despite CH showed a raise in adiposity in comparison to CC, alterations in the gestational period seem to be more harmful to offspring's body composition, since both HH and HC groups presented the highest levels of adiposity (Fig. 4b). Caloric intake was elevated in CH (20%), HH (21%) and HC (20%; Fig. 4c) as well as fasting glucose for the same groups in comparison to CC (1.6-, 1.7- and 1.7-fold, respectively) (Fig. 4d).

Serum CHOL and TAG levels were higher in HH in comparison to CC (1.3-fold). Furthermore, among the cross-fostered mice, HC showed higher CHO and TAG levels than CH (1.3- and 1.5-fold, respectively - Fig. 4e).

The relative expression of oxidative (*Cpt1a* and *Acadvl*) and lipogenic genes (*Agpat* and *Gpam*) in liver was assessed in crossfostered and unfostered offspring. Interestingly *Cpt1a* expression in HH was reduced (46%) compared to CC (Fig. 4f), suggesting reduction in fatty acid oxidation in this group. However, no differences in crossfostered groups were observed. However, *Acadvl* expression, another marker of fatty acid oxidation, was significantly reduced in unfostered HH (35%), as well as in crossfostering groups (CH and HC) (Fig. 4f).

Additionally, as showed in Fig. 4g, either *Agpat* or *Gpam*, both genes involved in triglycerides synthesis pathway, were increased in HH (62% and 33%, respectively) and CH (87% and 38%, respectively), but they were significantly higher in HC (164% and 62%, respectively), another indicative of the detrimental effect of diet on gestational period. HH presented an increasing in hepatic total lipid content (173%) despite HC have shown a tendency to increasing in this parameter when compared to CC and CH (Fig. 4h).

Interestingly, hepatic *miR-122* expression was significantly reduced in CH (2.2-fold), HH (1.5-fold) and HC (2.1-fold) compared to CC, while *mir-370* expression was significantly increased in CH (4.4-fold), HH (7.7-fold) and HC (8.9-fold) (Fig. 4i), corroborating the data showing that HFD feeding at gestational period leads to alterations in these miRNAs in offspring and also showing that excessive lipids consumption at lactation period can also lead to these miRNAs modulation. Moreover, *miR-122* expression in the liver of recently weaned offspring showed inversely correlation with serum TAG levels (Fig. 4j).

### Metabolic programming and hepatic miRNAs modulation by maternal HFD consumption in gestation and lactation persists into adult life of offspring

**Fig. 5:**
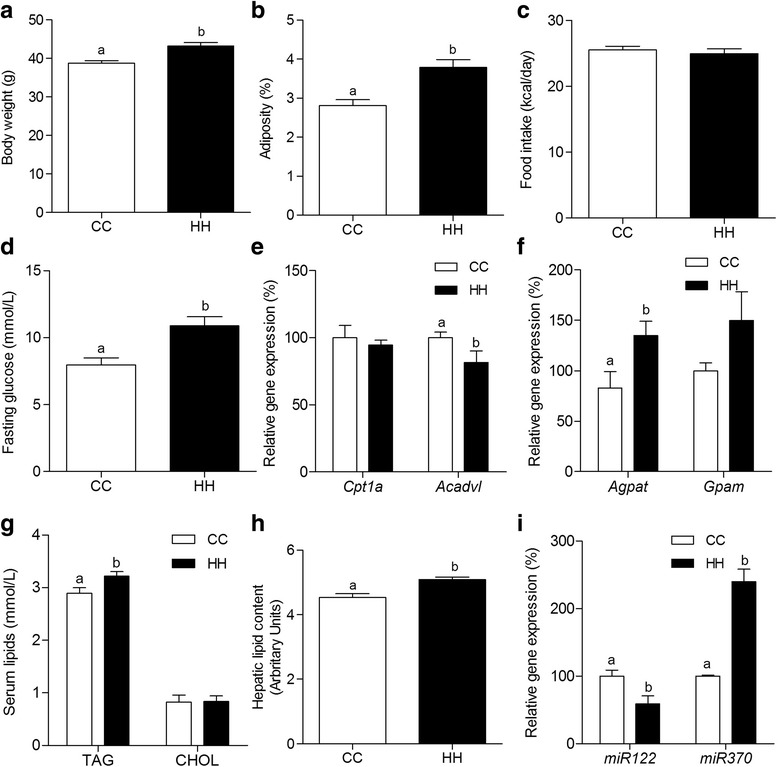
Metabolic programming and hepatic miRNAs modulation by maternal HFD consumption persists into adult life of offspring. Body weight (**a**), adiposity (**b**), caloric intake (**c**), fasting glucose (**d**), mRNA levels (qRT-PCR) of hepatic *Cpt1a* and *Acadvl* (**e**) and *Agpat* and *Gpam* (**f**), serum lipids (CHOL and TAG - **g**), total hepatic lipid content (**h**), microRNA level (qRT-PCR) of hepatic *miR-122* and *miR-370* (**i**) from adult offspring from CC and HH groups at d82. For relative gene expression analysis, β-Actin and U6snRNA were used as endogenous controls. Values are means (*n* = 5–8) + - SEM. Student's *t*-test was used in all analyses to compare CC and HH groups. Different letters indicate statistical significance between groups (*p* ≤ 0.05)

To investigate whether the metabolic phenotype and hepatic miRNAs modulations observed previously [25] and reported here in CC and HH d28 offspring would persist into adult life, we evaluated the same parameters at d82. Body composition of HH remained altered, since body weight (11.5%) and adiposity (35%) were higher in this group in comparison to CC (Fig. 5a and b, respectively), although food intake was not different between the groups (Fig. 5c). Fasting glucose was also higher in HH (1.4-fold) at d82 (Fig. 5d). Hepatic gene expression analysis showed that, besides *Cpt1*a did not differ, *Acadvl* expression was still decreased in HH (19% - Fig. 5e) and, moreover, *Agpat* expression remained highly increased (63%), although *Gpam* was not significantly different between CC and HH (Fig. 5f). CHOL levels were not changed, but TAG levels were higher (1.1-fold - Fig. 5g) and total hepatic lipids were increased in HH (1.1-fold - Fig. 5h).

Importantly, the modulation of hepatic miRNAs by maternal HFD consumption at gestation and lactation also persists to adult life, since HH showed lower *miR-122* (42%) and higher *miR-370* (139%) expression in comparison to CC at d82 (Fig. 5i).

### Maternal HFD consumption at gestation and lactation leads offspring to increased metabolic complications when exposed to a HFD in adult life

Finally, we evaluated whether an HFD exposure in early life and its consequent modulation of key hepatic miRNAs would increase HFD sensitivity in later life and negatively affect lipid and glucose metabolism in offspring, we used a model in which offspring from obese and lean dams were re-challenged to HFD.

At d42, we investigated some parameters of offspring prior to exposure to HFD in order to observe whether the phenotype of HH group remained different from CC group, as observed on d28. Compared to CC, HH were still heavier (10%) and presented higher fat mass (38%) (Additional file 2: Figure S2A and B, respectively), although caloric intake between groups was not different (Additional file 2: Figure S2C). HH presented impaired glucose homeostasis with more elevated fasting glucose (1.3-fold) and insulin (1.2-fold) than CC (Additional file 2: Figure S2D and E, respectively). Moreover, TAG levels were higher in HH, despite CHOL serum levels were not different between the groups (Additional file 2: Figure S2F).

**Fig. 6:**
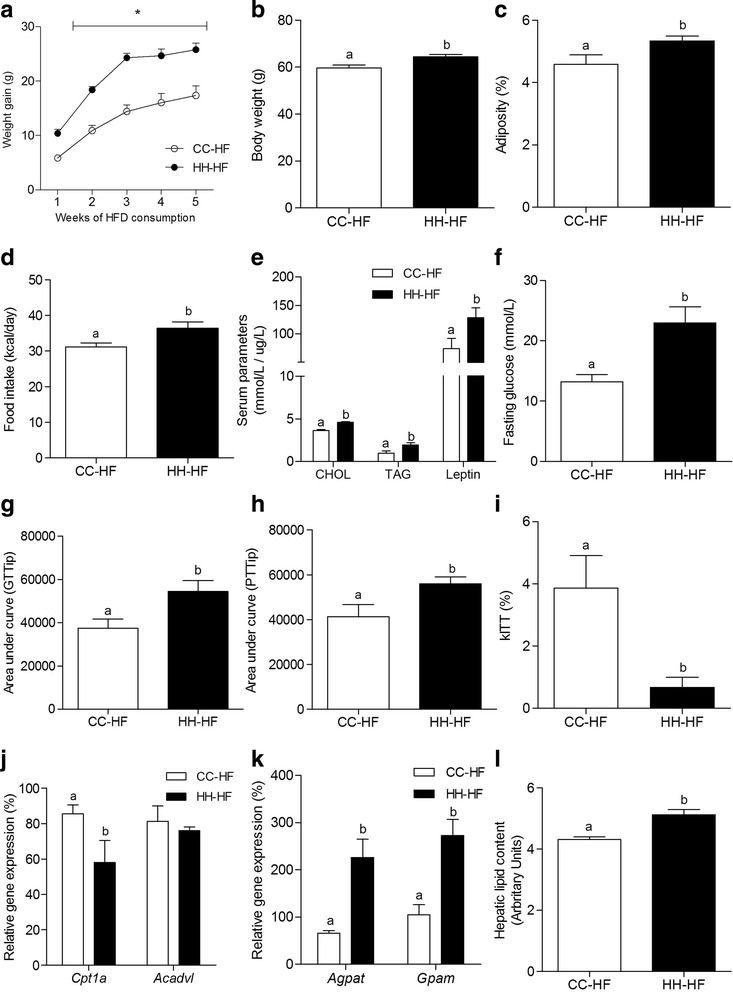
Maternal HFD consumption leads offspring to increased metabolic complications when exposed to HFD in adult life. Weight gain (**a**), body weight (**b**), adiposity (**c**), caloric intake (**d**), serum parameters (CHOL, TAG and leptin) (**e**), fasting glucose (**f**), area under curve (AUC) of GTT (**g**) and PTT (**h**), glucose clearance during ITT (**i**), mRNA levels (qRT-PCR) of hepatic *Cpt1a* and *Acadvl* (**j**) and *Agpat* and *Gpam* (**k**), total hepatic lipid content (**l**) from adult offspring after exposure to HFD for 40 days at d82 (CC-HF and HH-HF groups). For relative gene expression analysis, β-Actin was used as endogenous control. Values are means (*n* = 5–8) + - SEM. Student's *t*-test was used in all analyses to compare CC-HF and HH-HF groups. Different letters indicate statistical significance between groups (*p* ≤ 0.05)

After HFD exposure for over five weeks, HH–HF presented a significantly higher weight gain, as early as the first week of HFD consumption, and an increased total body mass (11%) in comparison to CC–HF (Fig. 6a and b, respectively). Adiposity was also increased in HH-HF (35%), as well as food intake (1.2-fold) (Fig. 6c and d, respectively).

Serum parameters reveals higher levels of CHOL, TAG, leptin and fasting glucose in HH-HF (Fig. 6e and f) and, moreover, tolerance tests showed that HH-HF presented disturbances in glucose homeostasis, since these group presented higher AUC in GTT (45%) and PTT (36%) and diminished glucose clearance as shown in kITT (5.7-fold - Fig. 6g, h and i, respectively).

Besides no differences were observed in *Acadvl*, *Cpt1a* expression was decreased in HH-HF liver (Fig. 6j) and, furthermore, *Agpat* and *Gpam* expression were markedly increased (242% and 161%, respectively - Fig. 6k). Additionally, HH-HF presented higher levels of hepatic total lipid content when in consumption of a HFD than CC-HF (18.5% - Fig. 6l).

## Discussion

Maternal consumption of HFD during critical developmental periods has been shown to lead to commitments on offspring's homeostasis and may result in metabolic disorders [11, 28]. We recently showed that maternal consumption of HFD during gestation and lactation activates pro-inflammatory pathways and unfolded protein response, impairs cholinergic anti-inflammatory pathway, modulates autophagy proteins and also affects lipid metabolism in offspring [25, 36–38]. Offspring from HFD-fed dams present downregulation of hepatic β-oxidation-related genes and upregulation of genes involved in lipid synthesis, and these alterations seem to be driven by the modulation of *miR-122* and *miR-370* levels, causing metabolic adaptations that lead to increased ectopic lipid accumulation in the liver of recently weaned mice [25].

Despite some studies have suggested that maternal macronutrients may be responsible for gene expression modulation in foetal offspring [39–41], we have not found studies showing that components of maternal diet could directly alter miRNAs expression in offspring. It is known that a HFD consumption increases lipids levels in serum, such as TAG, CHOL and free fatty acids (FFA) [25] and, moreover, maternal diet can directly affect breast milk composition [32–34, 42]. Therefore we hypothesized that the lipids consumed by the dams, specially SFA, could be responsible for *miR-122* and *miR-370* modulation in the liver of offspring through placental delivery during gestation and/or milk composition during suckling period.

As shown in the present study, treatment with palmitic acid, one of the most abundant SFAs in the human diet and blood, leads to a decrease in *miR-122* and increased *miR-370* levels in hepatocytes. It have been largely reported that SFAs play an important role in the development of insulin resistance by their directly regulation of inflammatory and metabolic pathways [15, 16, 43]. More recently, studies showed that several miRNAs are dysregulated in rodent models of diet-induced obesity, type 2 diabetes and NAFLD [44], reinforcing that excessive fat consumption, particularly SFAs, may be involved in miRNAs modulation that participate in the genesis of metabolic diseases. Importantly, in vitro analysis showed that miRNAs can be direct targets of dietary components, including fatty acids [19, 45]. Moreover, Nie and colleagues (2014) induced lipid accumulation in human hepatocyte cell line using oleic acid. In this steatotic hepatocyte model, *miR-122* expression was downregulated and, importantly, transfection with *miR-122* mimic significantly reduced lipids within the hepatocytes [46].

After confirming that the in vitro treatment with palmitate was able to reduce *miR-122* and increase *miR-370* expression, we aimed to investigate the independent contribution of maternal excessive lipids consumption at gestational or lactation periods on the modulation of these miRNAs, since only few studies have investigated the relative contribution of pre- or post-natal maternal HFD consumption to metabolic phenotype in offspring. Sun and colleagues [47] showed that maternal HFD consumption during the suckling period has a high influence on leptin signaling in offspring. On the other hand, Cerf and colleagues [48] showed that neonates exposed to HFD only during foetal development presented reduced volume and number of β-cells, accompanied by sustained and irreversible hyperglycaemia during adulthood.

Here, we showed that pups from HFD-fed dams presents lower body weight and LIO at birth (d0), in comparison to pups from control dams. Similar studies have shown higher body weight and macrosomia in newborns from obese dams [49, 50] however, some authors have shown reduced body weight in offspring at d0 using models of maternal hyperglycemia and transient hyperinsulinemia. In our model, HFD-fed dams presented higher insulin levels prior to mating when compared to control dams and, although this hormone does not cross the placental barrier, some authors suggest that maternal hyperinsulinemia may lead to placental disorders and that this occurrence may be related to the delayed foetus development [51–53].

Interestingly, besides the lower body weight, hepatic lipid synthesis seems to be increased in offspring from HFD-fed dams at birth, as suggested by liver *Agpat* and *Gpam* levels and, in contrast, genes related to fatty acid oxidation (*Cpt1a* and *Acadvl*) are reduced in their liver. There are several evidences in humans indicating that maternal obesity leads to increased fuel availability for the foetus and may drive to increased hepatic fat storage [54]. Maternal nutritional overload and placental transfer is a challenge to foetal development and, as suggested by Brumbaugh and Friedman [54], until final gestational phases, subcutaneous fat is not available to act as a storage buffer so, under dietary excesses, the foetal liver, among another organs, becomes a fat deposit. McCurdy and co-workers [55] showed, in non-human primates, an increase in hepatic TAG and elevated hepatic expression of gluconeogenesis key genes in foetus from HFD-fed dams at gestational third trimester and the maternal resistance to obesity development did not reduce the effects of the HFD-consumption on hepatic metabolism of the fetuses. Therefore, these findings suggest that maternal HFD consumption during pregnancy can modulate lipid metabolism in offspring, favoring fat deposition.

As we have speculated, *miR-122* and *miR-370* may be involved in the impairments of lipid homeostasis of newborns from HFD-fed dams, since we find here that these miRNAs are modulated at d0, as early as the hepatic enzymes involved in lipid metabolism. Furthemore, maternal FFA at gestation correlated directly with *miR-370* and inversely with *miR-122* in the liver of newborns, thus reinforcing the hypothesis that maternal lipids that cross placental barrier are able to modulate miRNAs expression of offspring.

It have been shown that mice lacking the gene encoding *miR-122a* are viable, but they develop hepatosteatosis, NASH and fibrosis and present hepatic infiltration of inflammatory cells that produce pro-tumorigenic cytokine, including IL-6 and TNFα, thus leading to the development of hepatocellular carcinoma [22, 26, 56]. Besides, Hsu and colleagues (2012) showed that knockout mice for *miR-122* in liver present higher expression of several hepatic enzymes involved in TAG synthesis, including *Agpat* [56]. Additionally, Xu and colleagues showed that inhibition of *miR-370* led to downregulation of pro-inflammatory cytokines, suggesting that this miRNA plays a pro-inflammatory role and may be related to hepatic damage [57]. Although the relationship between the levels of *miR-122* and *miR-370* is still subject to debate [14, 19], we showed that there is an inverse expression of these miRNA in our model at different ages. Thus, considering the impact of lipid metabolism and inflammatory signaling on the development of NAFLD, maternal HFD consumption during the gestational period could be considered an important risk factor for liver diseases in offspring.

Using the cross-fostering model, we were able to further confirm the importance of maternal HFD consumption during gestation to hepatic lipid metabolism and also investigate whether the suckling period would exert the same effect. Surprisingly, *miR-122* and *miR-370* levels seem to be also modulated by maternal milk, since pups from control dams suckled by HFD-fed dams showed a decrease in *miR-122* and increased expression of hepatic *miR-370* as well as upregulation of TAG synthesis markers (*Agpat* and *Gpam*) and downregulation of a fatty oxidation marker (*Acadvl*). Regardless of the negative effects of HFD consumption at gestational period seem to be more pronounced, in general, the impairments in lipid homeostasis observed at gestation are also present at the suckling period by itself. Although we did not evaluate milk composition, previous studies demonstrated that HFD consumption and mainly the availability of plasma fatty acid for uptake by the mammary gland alters the fatty acid composition and content, specially medium chain fatty acids secreted in milk of human and rat [42, 58, 59], and may exert effects on the fat accumulation in the neonatal liver.

Besides, offspring from HFD-dam presents altered serum lipid levels [25] and here, offspring's serum TAG correlated directly with *miR-370* and inversely with *miR-122* hepatic expression.

Given that we showed that excess maternal lipids at both gestational and lactation periods are independently able to alter hepatic miRNAs in newborn and recently weaned offspring, respectively, that may lead to impaired lipid metabolism, the next step was to investigate whether these modulation would persist into adult life. As shown here and previously, offspring exposed to maternal HFD at gestation and lactation still present, at d82, increased body weight, adiposity, fasting glucose and fat accumulation within the liver. Interestingly, adult offspring from HFD-fed dam still shows decreased *miR-122* and increased *miR-370* expression in the liver. These findings suggest that epigenetic modifications at pre and recent post-natal life are persistent. Thorn and colleagues (2014) also showed that, in non-human primates, maternal insulin resistance induced by HFD consumption at developmental stages negatively and irreversible affects hepatic immune system and development of *de novo* lipogenic pathways [60]. For these reasons, authors believes that exposure to excess maternal lipids at critical development periods may be considered the "first hit" for the pathogenesis of liver diseases [60, 61].

The "two hits" or "multiple hits" hypothesis was postulated as an attempt to explain the mechanisms underlying the progression of NAFLD to more aggressive liver diseases, such as NASH and fibrosis. Ectopic accumulation of fat in the liver, the steatosis, was considered the "first hit", and authors suggested that a second hepatic insult, such as oxidative stress or drugs, would be necessary to lead to chronical inflammation and NAFLD progression [62, 63]. Nowadays, it is hypothesized that the obesity and lipid overload in intra-uterine environment could program the foetal liver and trigger the "first hit" and, afterwards, a high-fat diet consumption in post-natal life would be enough to lead to hepatic inflammation and to liver diseases progression [61].

In this context, we showed here that offspring challenged with maternal lipid overload in pre-natal life and through lactation present a more deleterious response when exposed to a HFD in adult life, by showing an increase in weight gain, adiposity and serum parameters, such as CHOL, TAG, leptin and fasting glucose and, moreover, exacerbated glucose homeostasis disturbances and lipid accumulation in the liver, factors that may lead to inflammatory pathways activation. We showed recently that glucose homeostasis disturbances observed in adult offspring re-challenged to HFD may occur due to an imbalance in insulin signaling in peripheral tissues (such as visceral adipose tissue) and hypothalamus. Additionally a failure in glucose production blockade in the liver was also observed [64]. Therefore, since insulin resistance plays a central role in the metabolic syndrome and the ectopic lipid accumulation in the liver has been considered the hepatic manifestation of this condition [4, 5], our results suggest that offspring from obese dams that are re-exposed to a HFD in adult life could present a metabolic syndrome-like phenotype.

Besides others studies also had shown these increased metabolic disturbances in adult offspring from obese dams exposed to a HFD in adult [65], to our knowledge, the present study was the first to show that maternal diet leads to persistent modulation of key liver miRNAs that may be involved in the development of insulin resistance and NAFLD. Thus, the attention to maternal diet during gestation and lactation seems to be of great importance to avoid permanent changes during foetal development that could contribute to the development of obesity and its comorbidities in later life.

## Conclusions

In summary, maternal HFD consumption during gestation and lactation affect the metabolic phenotype in the offspring. The presence of fatty acids in maternal blood and milk seem to be responsible for modulating the expression of liver *miR-122* and *miR-370*, which are involved in liver homeostasis. These findings provide important information regarding the effects of maternal nutrition, especially during gestation, on the metabolic phenotype of offspring. However, further studies are necessary to understand how nutritional intervention during gestational and lactation periods can contribute to reduce metabolic damage in offspring.

## Additional files

Additional file 1: Figure S1. Antropometric and biochemical parameters of dams at gestation and lactation. Body weight (A), adiposity (B), fasting glucose (C), serum insulin (D) and lipids (CHOL and TAG - E) and total hepatic lipid content (F) of control (C-D) and HFD (HF-D) dams in the gestational day 12, and lactational day 15. Values are means (*n* = 3-6) + - SEM. Student's *t*-test was used in all analyses to compare C-D and HF-D groups in each period (gestation or lactation). Different letters indicate statistical significance between groups (*p* ≤ 0.05). (JPG 667 kb)
Additional file 2: Figure S2. Characterization of d42 offspring from control and obese dams prior to HFD exposure. Body weight (A), adiposity (B), caloric intake (C), fasting glucose (D), serum insulin (E) and lipids (CHOL and TAG - F), in d42 CC and HH groups. Values are means (*n* = 4-8) + - SEM. Student's *t*-test was used in all analyses to compare CC and HH groups. Different letters indicate statistical significance between groups (*p* ≤ 0.05). (JPG 390 kb)

## Abbreviations

Acadvl: Acyl-CoA dehydrogenase very long-chain
Agpat: 1-acylglycerol-3-phosphate acyltransferase
CHOL: Cholesterol
Cpt1a: Carnitine palmitoyltransferase 1a
FFA: Free fatty acid
Gpam: Glycerol-3-Phosphate acyltransferase mitochondrial
HFD: High-fat diet
IL-6: Interleukin 6
miR/miRNA: microRNA
NAFLD: Non alcoholic fatty liver disease
NASH: Non alcoholic steatohepatitis
NEFA: Non-esterified fatty acid
Scd1: Stearoyl-CoA desaturase 1
SFA: Saturated fatty acid
TAG: Triacylglycerol
TNFα: Tumor necrosis factor α
