# Supplementary figures and images for: Lipid overload during gestation and lactation can independently alter lipid homeostasis in offspring and promote metabolic impairment after new challenge to high-fat diet
Source: Nutr Metab (Lond). 2017 Feb 20;14:16. doi: 10.1186/s12986-017-0168-4 (PMC5319047; doi:10.1186/s12986-017-0168-4)

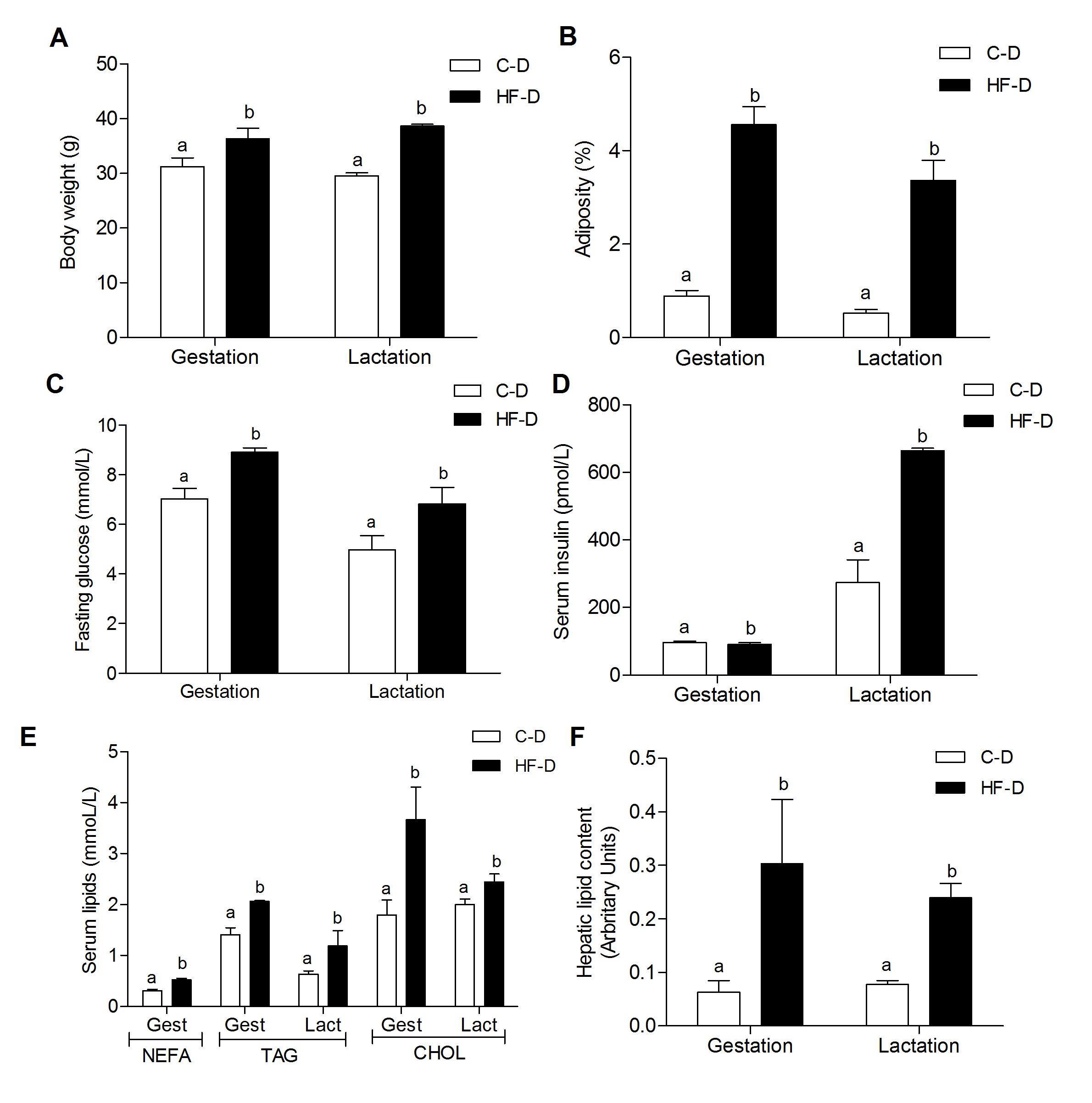

Supplement: Additional file 1: Figure S1. — Antropometric and biochemical parameters of dams at gestation and lactation. Body weight (A), adiposity (B), fasting glucose (C), serum insulin (D) and lipids (CHOL and TAG - E) and total hepatic lipid content (F) of control (C-D) and HFD (HF-D) dams in the gestational day 12, and lactational day 15. Values are means (n = 3-6) + - SEM. Student's t-test was used in all analyses to compare C-D and HF-D groups in each period (gestation or lactation). Different letters indicate statistical significance between groups (p ≤ 0.05). (JPG 667 kb) [file 12986_2017_168_MOESM1_ESM.jpg]

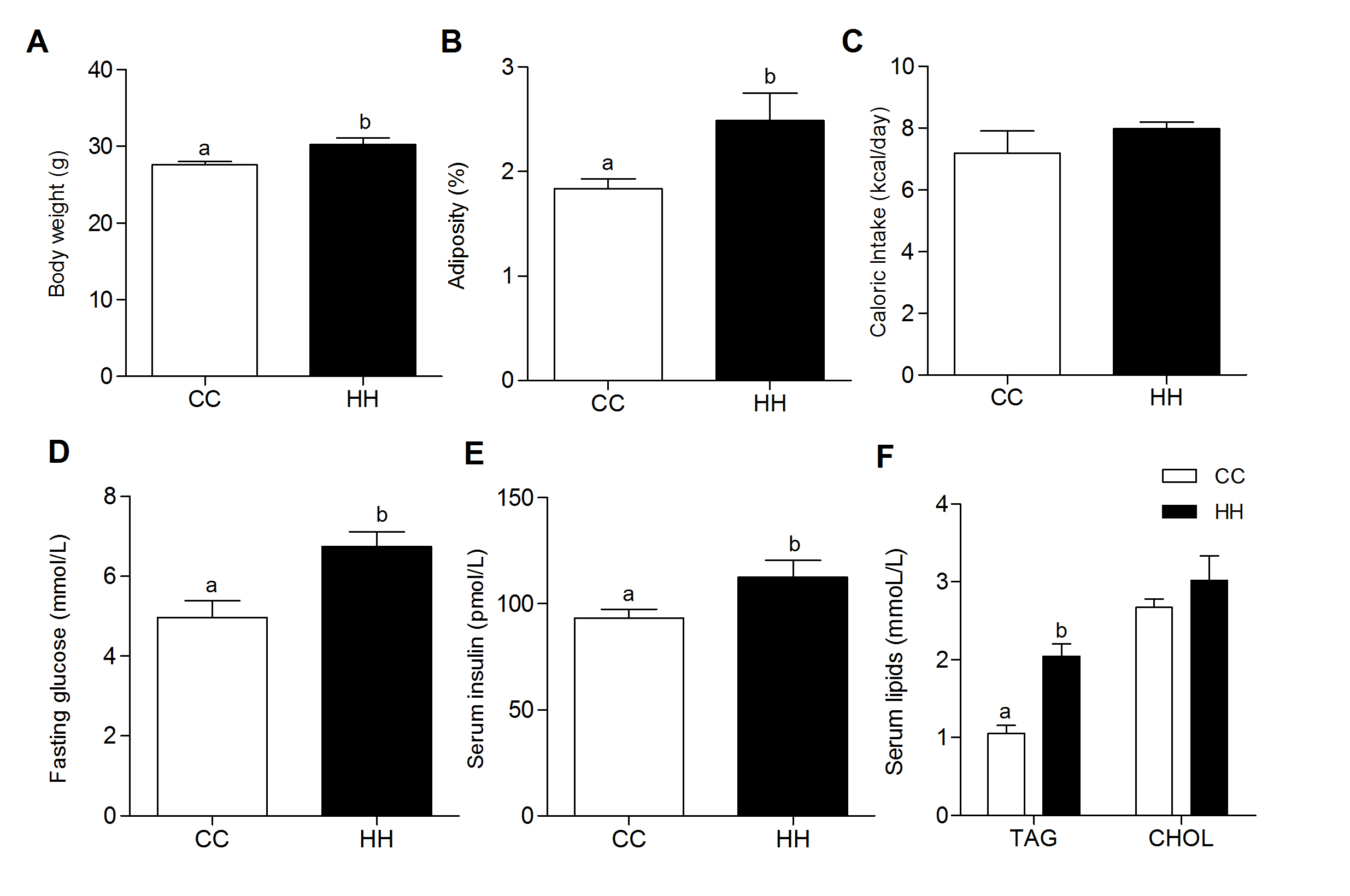

Supplement: Additional file 2: Figure S2. — Characterization of d42 offspring from control and obese dams prior to HFD exposure. Body weight (A), adiposity (B), caloric intake (C), fasting glucose (D), serum insulin (E) and lipids (CHOL and TAG - F), in d42 CC and HH groups. Values are means (n = 4-8) + - SEM. Student's t-test was used in all analyses to compare CC and HH groups. Different letters indicate statistical significance between groups (p ≤ 0.05). (JPG 390 kb) [file 12986_2017_168_MOESM2_ESM.jpg]
